# Active Shell Engineering for Efficient Cascade Triplet Energy Transfer in Lanthanide Heterostructures

**DOI:** 10.1002/anie.2017963

**Published:** 2026-02-22

**Authors:** Zhao Jiang, Alasdair Tew, Xinjuan Li, Huangtianzhi Zhu, Yunzhou Deng, Caterina Ducati, Zhongzheng Yu, Akshay Rao

**Affiliations:** ^1^ Cavendish Laboratory University of Cambridge Cambridge UK; ^2^ Department of Materials Science and Metallurgy University of Cambridge Cambridge UK

**Keywords:** heterostructure, lanthanide‐doped nanoparticle, molecular sensitization, surface passivation, triplet energy transfer

## Abstract

Lanthanide‐doped nanoparticles (LnNPs) exhibit unique optical properties but suffer from severe surface quenching and weak absorption that fundamentally limit their performance. Here, we demonstrate a breakthrough cascade triplet energy transfer (TET) mechanism in precisely engineered NaYbF_4_@Ca_0.8_F_2_:Nd_0.2_@9‐anthracenecarboxylic acid (ACA) heterostructures. This core/active shell/organic molecule configuration combines both molecular sensitization with surface passivation, transforming conventional inert barriers into functional energy conduits. We explore in detail the synthetic conditions required to grow not just optimally active shells but also how best to assemble the organic ligands on the surface of core‐shell LnNPs. Systematic shell thickness optimization (0.8–4.6 nm) reveals an optimal shell thickness of ∼2.0 nm. When coupled with an appropriate ligand exchange strategy, we achieve a remarkable 1200‐fold emission enhancement compared to bare cores. Comprehensive spectroscopic investigations confirm near‐unity TET efficiency and reveal the cascade TET mechanism utilizing Nd^3+^ ions as energy intermediates to maximize the Yb^3+^ emission. Thus, our mechanism and heterostructure design present one of the most promising synthetic strategies to overcome the existing limitations of traditional LnNPs, establishing new paradigms for high‐performance heterostructures with broad applications in bioimaging, photon conversion, and optoelectronic devices.

## Graphical abstract

Active shell engineering resolves the trade‐off between surface passivation and molecular sensitization in lanthanide nanoparticles. NaYbF_4_@Ca_0.8_F_2_:Nd_0.2_@ACA heterostructures transform inert shells into energy conduits, enabling cascade triplet transfer with near‐unity efficiency. Optimal shell thickness (∼2 nm) simultaneously achieves surface protection and efficient energy relay, establishing design paradigms for high‐performance luminescent materials.

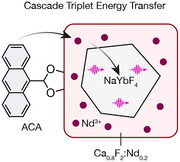

## 1 Introduction

Lanthanide‐doped nanoparticles (LnNPs) exhibit remarkable optical properties, including sharp emission profiles, extended luminescence lifetimes, and exceptional photostability. These characteristics have established LnNPs as valuable materials for advanced applications spanning upconversion [1, 2, 3], laser technology [4, 5, 6], infrared detection [7], bioimaging [8, 9, 10, 11], optogenetics [12, 13], and security encryption [14, 15]. Despite their potential, two challenges persistently limit LnNPs performance: surface quenching and weak absorption. Surface quenching occurs through non‐radiative relaxation pathways at surface defects (incomplete coordination, dangling bonds) and vibrational coupling to high‐energy bonds (O─H, N─H) in surface ligands and solvent molecules [16, 17], while weak absorption results from the parity‐forbidden nature of 4f‐4f electronic transitions in lanthanide ions, leading to inherently low excitation efficiency [18, 19, 20]. Together, these factors significantly compromise the overall optical performance of LnNPs.

Fabrication of heterostructures is one of the most effective strategies to overcome these limitations [20]. The fabrication of core‐shell heterostructures with inert heterogeneous shell preventing cation intermixing has recently been proven to be more effective to enhance the downconversion efficiency than the homogenous shell structure [21]. Organic–inorganic heterostructures have also been developed to simultaneously address the weak absorption of Ln^3+^ by incorporating molecular sensitizers with much higher absorption cross section (at least four orders of magnitude higher than that of LnNPs) [22, 23], which serve as antennas to enhance photon harvesting. However, these approaches present an inherent contradiction. While thick inert shells effectively suppress surface quenching, they hinder efficient energy transfer from external sensitizers to internal emitters by increasing the spatial separation as the energy transfer efficiencies are very sensitive to the distance between donor and acceptor.

Recent investigations have revealed the critical importance of triplet excitons in molecular sensitization of LnNPs [24]. LnNPs can enhance the intersystem crossing (ISC) rate of dye molecules attached onto the surface of LnNPs, facilitated by spin‐exchange coupling with the unpaired electrons of the Ln^3+^ ions, thereby rapidly converting singlet excitons to triplet excitons [24, 25, 26, 27]. The triplet excitons can be transferred to the appropriate energy levels of Ln^3+^ with high efficiencies [25]. These findings highlight the significance of triplet‐exciton mediated energy transfer mechanisms [24, 25, 27, 28, 29, 30], which can exceed the efficiency of singlet energy transfer mediated by Förster resonance energy transfer (FRET) due to the weak dipole of Ln^3+^ ions. The advantages of triplet states, including long lifetimes, better energy matching with lanthanide levels, and minimal competing decay pathways, make them ideal for efficient sensitization of LnNPs [31, 32, 33]. However, this also presents a fundamental challenge as it has been shown that the triplet energy transfer (TET) processes require short‐range Dexter‐type interactions with substantial orbital overlap [27]. Consequently, developing strategies that combine molecular triplet sensitization with surface passivation via thicker shells remains a huge challenge in this field.

**FIGURE 1:**
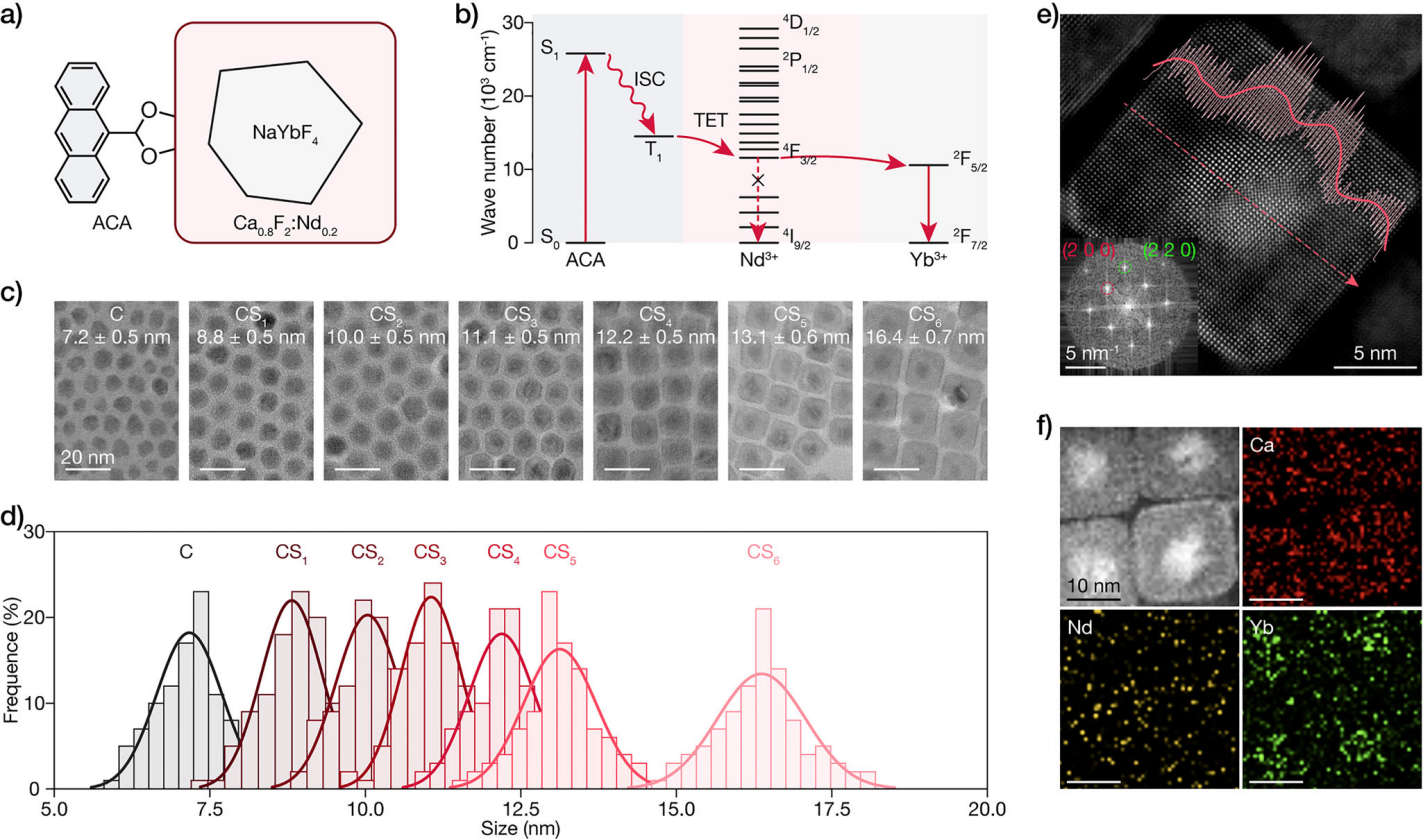
Core/active‐shell architecture design and structural characterization. a) Schematic illustration of the NaYbF_4_@Ca_0.8_F_2_:Nd_0.2_@ACA hybrid system. b) Energy level diagram illustrating the proposed energy transfer pathway from ACA molecules through Nd^3+^ in the shell to Yb^3+^ in the core via triplet states of ACA. c) TEM images of NaYbF_4_ core and NaYbF_4_@Ca_0.8_F_2_:Nd_0.2_ core‐shell NPs with increasing shell thickness. d) Size distribution histograms of the images showed in c). e) HAADF‐STEM image of sample CS_6_, with the signal intensity profile (red curve) along the dotted line revealing the core‐shell interface structure. The inset shows the fast Fourier transform pattern indicating the crystallinity of the sample. f) Energy‐dispersive x‐ray spectroscopy elemental mapping of sample CS_6_ confirming the spatial distribution of Ca (red), Nd (yellow), and Yb (green) elements.

In this work, we report the design of core/active‐shell/organic molecule heterostructures as shown in Figure 1a, based on NaYbF_4_@Ca_0.8_F_2_:Nd_0.2_ LnNPs and 9‐anthracenecarboxylic acid (ACA). Figure 1b shows the schematic illustration of the efficient cascade pathways in our heterostructures, utilizing Nd^3+^ ions as energy intermediates and involving TET from triplet excitons of ACA (T_1_ ≈ 1.83 eV) [34] to Nd^3+ 4^F_3/2_ (∼11,500 cm^−1^), and then Nd^3+^ to Yb^3+ 2^F_5/2_ (∼10,200 cm^−1^), with optimal energy gaps of ∼3,260 and ∼1,300 cm^−1^, respectively. We thus maximize the near infrared emission from Yb^3+^ (^2^F_5/2_ → ^2^F_7/2_) with a long lifetime of 2.3 ms. We would like to highlight that both processes can achieve near unity efficiencies. By systematically varying the shell thickness from 0.8 to 4.6 nm, we identify an optimal shell thickness (∼2.0–2.5 nm) at which emission enhancement reaches a remarkable 1200‐fold when sensitized by ACA. Through comprehensive spectroscopic investigations including steady‐state, time‐resolved, and transient absorption (TA) measurements, we elucidate the mechanism of energy transfer across the organic–inorganic interface and through the active shell. Our findings reveal that the Nd^3+^‐doped active shell plays a pivotal role in facilitating efficient energy capture from triplet states of organic sensitizers and transferring to the Yb^3+^ emitters. These insights establish design principles for molecule‐sensitized lanthanide nanomaterials with shells to obtain enhanced optical performance.

## 2 Results and Discussion

### 2.1 Heterostructures With Active Shell

Our core/active‐shell design employs a NaYbF_4_@Ca_0.8_F_2_:Nd_0.2_ architecture that simultaneously optimizes energy transfer and surface passivation. Unlike typical LnNPs that require low doping concentrations to prevent cross‐relaxation and concentration quenching, Yb^3+^ offers a unique advantage through its simple two‐level energy structure (^2^F_7/2_ and ^2^F_5/2_). Our experiments demonstrate that NaYbF_4_ with 100% Yb^3+^ content exhibits excellent luminescence performance, with emission intensities increasing proportionally with Yb^3+^ concentration and lifetime values comparable to those of low‐concentration samples (Figure S1). This high Yb^3+^ loading provides higher emitting center density, enhancing brightness beyond conventional low‐doping strategies. We thus selected NaYbF_4_ as the core emitter and incorporated Nd^3+^ into the shell to enable efficient energy transfer (Figure 1a). The Nd^3+^ doping concentration of 20% was optimized to balance energy relay efficiency with shell quality as higher concentrations lead to morphological deterioration and enhanced cross‐relaxation losses (Figures S2 and S3). CaF_2_ was chosen instead of homogeneous NaLnF_4_ shells to prevent cation intermixing at the core‐shell interface [21], ensuring enhanced passivation while maintaining efficient energy transfer. The efficient energy transfer from Nd^3+ 4^F_3/2_ level to the ^2^F_5/2_ level of core Yb^3+^ ions is facilitated by excellent energy level matching between these lanthanide ions, with an optimal energy gap of ∼1,300 cm^−1^ as confirmed by their absorption spectra (Figure S4). This energy gap enables efficient forward transfer while being sufficiently large to prevent thermal back‐transfer at room temperature (kT ≈ 207 cm^−1^). This energy transfer proceeds unidirectionally from Nd^3+^ to Yb^3+^, minimizing energy leakage while ensuring robust core passivation.

We then synthesized NaYbF_4_ core and a series of NaYbF_4_@Ca_0.8_F_2_:Nd_0.2_ core‐shell NPs with precisely controlled shell thicknesses and characterized their structural and optical properties. To ensure batch‐to‐batch consistency, all core NPs were synthesized following a strictly controlled protocol to maintain similar average sizes. Figures 1c and S5 show the morphological evolution and size distribution. The cubic NaYbF_4_ core NPs (C) were synthesized using a modified co‐precipitation method [35], with the cubic phase selected over hexagonal to reduce lattice mismatch with the shell. The core NPs exhibited irregular polyhedral morphology with an average diameter of 7.2 nm. As the Ca_0.8_F_2_:Nd_0.2_ shell thickness increased from CS_1_ (8.8 nm) to CS_6_ (16.4 nm), morphology gradually evolved from irregular polyhedrons to well‐defined hexagons and eventually to squares, characteristic of CaF_2_ crystal structure [36]. The core‐shell structure is clearly visible in TEM images, with darker contrast in the center corresponding to the higher atomic number Yb‐containing core, and lighter contrast of the Ca‐based shell. Size distribution histograms confirm uniform shell growth with progressively increasing diameters (Figure 1d).

The core‐shell architecture was further verified by high‐angle annular dark‐field scanning transmission electron microscopy (HAADF‐STEM) (Figures 1e and S6). The core region containing high‐Z Yb atoms (*Z* = 70) appears significantly brighter than the shell region dominated by lower‐Z Ca atoms (*Z* = 20), with Nd (*Z* = 60) as dopant. The signal intensity profile (red curve) along the dotted line clearly demonstrates this contrast, providing direct evidence of the core‐shell heterostructure. Interestingly, the fast Fourier transform (FFT) pattern reveals only one set of diffraction spots, suggesting a single‐crystalline structure. This reflects the remarkable structural similarity between cubic NaYbF_4_ and CaF_2_, which share the same space group (Fm‐3m) and have nearly identical d‐spacings that cause their diffraction spots to overlap in the FFT pattern (Table S1). Energy‐dispersive x‐ray spectroscopy (EDS) elemental mapping (Figure 1f) demonstrates Yb (green) compartmentalization in the core, while Ca (red) predominates in the shell. The Nd (yellow) signal appears less intense due to its relatively low dopant concentration (20%), but its distribution within the shell region remains discernible. This elemental mapping verifies successful synthesis of our designed NaYbF_4_@Ca_0.8_F_2_:Nd_0.2_ core/active‐shell architecture.

### 2.2 Shell Thickness‐Dependent Surface Passivation

Prior to photoluminescence (PL) measurements, we recorded absorption spectra of all samples (Figure S7). The spectra reveal characteristic Nd^3+^ absorption bands in the shell, with intensities increasing systematically with shell thickness. To ensure accurate comparison across samples, concentrations were calibrated based on Yb^3+^ absorption at 980 nm.

**FIGURE 2:**
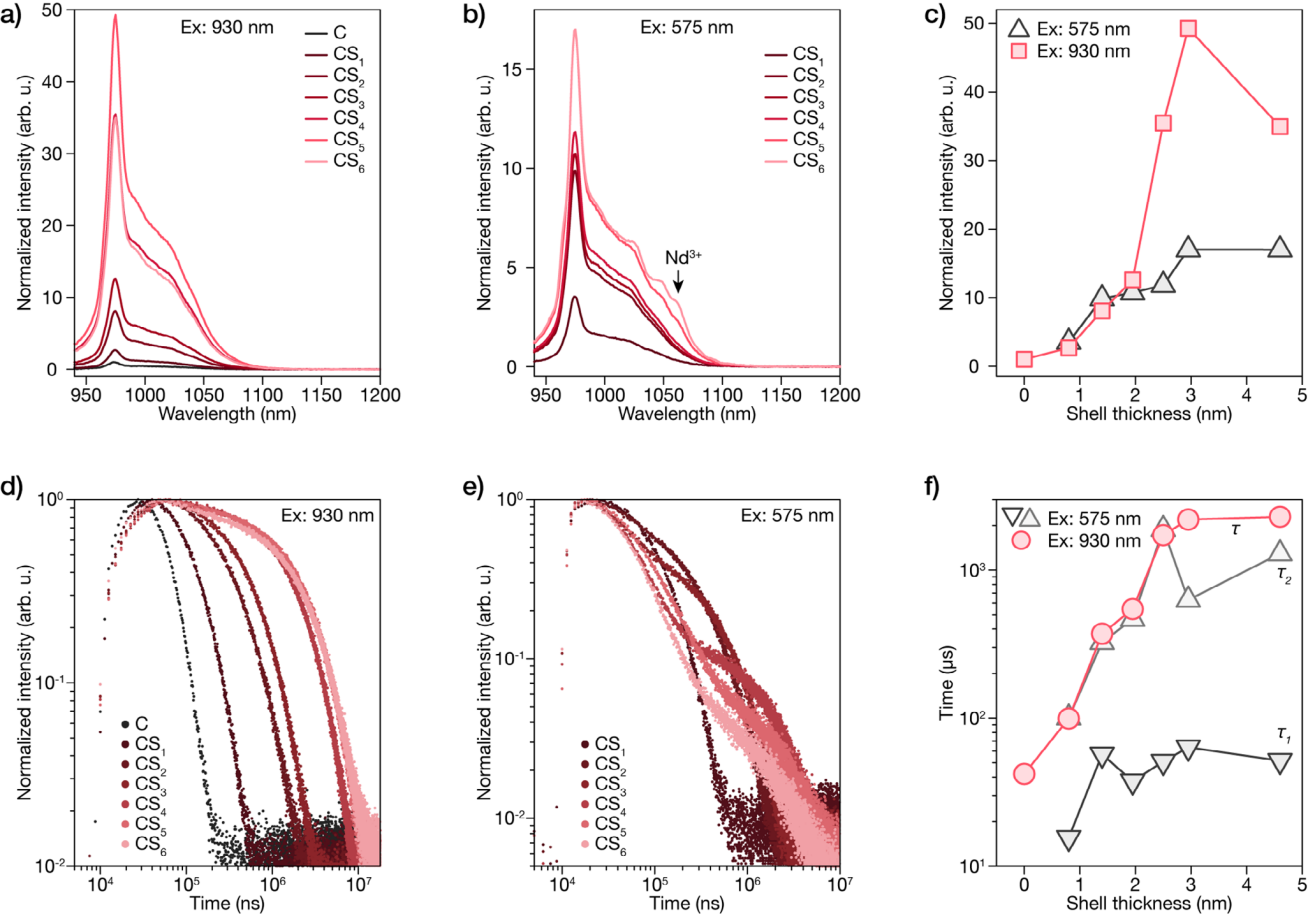
Enhanced Yb^3+^ emission in core/active‐shell LnNPs with increasing shell thickness. a) Emission spectra of NaYbF_4_ core (C) and NaYbF_4_@Ca_0.8_F_2_:Nd_0.2_ core‐shell LnNPs (CS_1_–CS_6_) under direct 930 nm excitation of Yb^3+^. b) Emission spectra of the same series of NPs under 575 nm excitation of Nd^3+^ in the shell. c) Integrated emission intensity as a function of shell thickness under both excitation wavelengths (575 and 930 nm), all data normalized to the emission of core NPs under 930 nm excitation. d) Photoluminescence decay curves of Yb^3+^ emission at 980 nm under 930 nm excitation of Yb^3+^. e) Photoluminescence decay curves of Yb^3+^ emission at 980 nm under 575 nm excitation of Nd^3+^. f) Fitted time constants from single‐exponential and bi‐exponential fitting of decay curves in d) and e), respectively.

We assessed the effect of shell thickness on optical properties by measuring PL spectra and decay profiles under different excitation wavelengths. Figure 2a shows emission spectra under direct 930 nm Yb^3+^ excitation, normalized to core sample intensity. Yb^3+^ emission at 980 nm progressively intensifies with shell thickness, reaching a ∼50‐fold enhancement for CS_5_. This substantial enhancement stems from effective surface passivation by the Ca_0.8_F_2_:Nd_0.2_ shell, which mitigates non‐radiative surface quenching. Notably, sample CS_6_ shows slight emission decrease despite greater shell thickness. Since crystalline quality remains excellent throughout the series (confirmed by STEM analysis), this non‐monotonic trend likely results from competing interface effects. At excessive shell thicknesses, weak Yb^3+^ to Nd^3+^ back‐energy transfer or cross‐relaxation processes may become significant, slightly diminishing overall emission efficiency. Figure 2b presents emission spectra under 575 nm excitation, which selectively excites shell Nd^3+^ ions. All spectra were corrected using excitation spectra (Figure S8) and normalized to the core sample under 930 nm excitation. Note that the core sample is absent from this dataset as it lacks Nd^3+^ ions to absorb 575 nm excitation. Emission intensity increases monotonically with shell thickness, since thicker shells accommodate more Nd^3+^ ions that harvest excitation energy and transfer it to core Yb^3+^ ions. Spectral features beyond 1050 nm differ from those in Figure 2a, exhibiting additional shoulders attributed to residual Nd^3+^ emission around 1060 nm. The negligible intensity of these Nd^3+^ features relative to the dominant Yb^3+^ emission confirms a high Nd^3+^ to Yb^3+^ energy transfer efficiency approaching unity.

Figure 2c shows the integrated emission intensities versus shell thickness under both excitation wavelengths. Under 930 nm excitation, emission intensity increases sharply up to ∼3 nm shell thickness, then plateaus with slight decrease for the thickest shell. This suggests optimal surface passivation at 3–3.5 nm thickness, beyond which additional shell material provides minimal benefit and may even introduce competing energy pathways. In contrast, under 575 nm excitation, the emission intensity continues to increase with shell thickness, though at a reduced rate beyond 3 nm. Even with the thickest shell, Nd^3+^ excitation yields substantially lower emission than direct Yb^3+^ excitation (Figures 2c and S8).

Time‐resolved PL measurements provide deeper understanding on energy transfer kinetics. Figures 2d,e and S9 display Yb^3+^ emission decay curves under 930 and 575 nm excitation. Under 930 nm direct excitation, decay profiles were fitted using single‐exponential functions, while under 575 nm excitation through Nd^3+^, bi‐exponential functions were required to extract fast (τ_1_) and slow (τ_2_) components and their relative weights (w_1_ and w_2_) (Table S2). Figure 2f summarizes the fitted time constants versus shell thickness. Under 930 nm excitation, the single decay component shows a progressive increase from 42 µs to 2.3 ms with increasing shell thickness, reflecting improved surface passivation that reduces non‐radiative decay pathways. In contrast, under 575 nm excitation, the decay dynamics are more complex, requiring bi‐exponential fitting due to the multi‐step energy transfer process from Nd^3+^ to Yb^3+^. Both τ_1_ and τ_2_ components are consistently shorter than the single component under direct 930 nm excitation, indicating accelerated decay stemming from additional energy transfer steps and energy migration barriers at the core‐shell interface. This extended pathway increases energy dissipation probability at the core‐shell interface. These contrasting decay dynamics reveal that while thicker shells improve surface passivation for direct excitation, they introduce longer energy migration pathways for sensitized excitation. The bi‐exponential decay kinetics under 575 nm excitation further confirm the Nd^3+^ to Yb^3+^ energy transfer process, validating a key step in our proposed cascade TET mechanism.

### 2.3 Ligand Exchange Optimization for Triplet Sensitization

To create our complete heterostructures, we add active organic ligands to our core‐shell NPs (characterized above). In order to achieve efficient TET in this full hybrid system, we systematically studied the ligand exchange conditions using CS_3_ NPs as the model substrate. The ligand exchange process involves three critical parameters: ligand concentration, reaction time, and washing cycles, each of which may influence the surface coverage and energy transfer efficiency.

**FIGURE 3:**
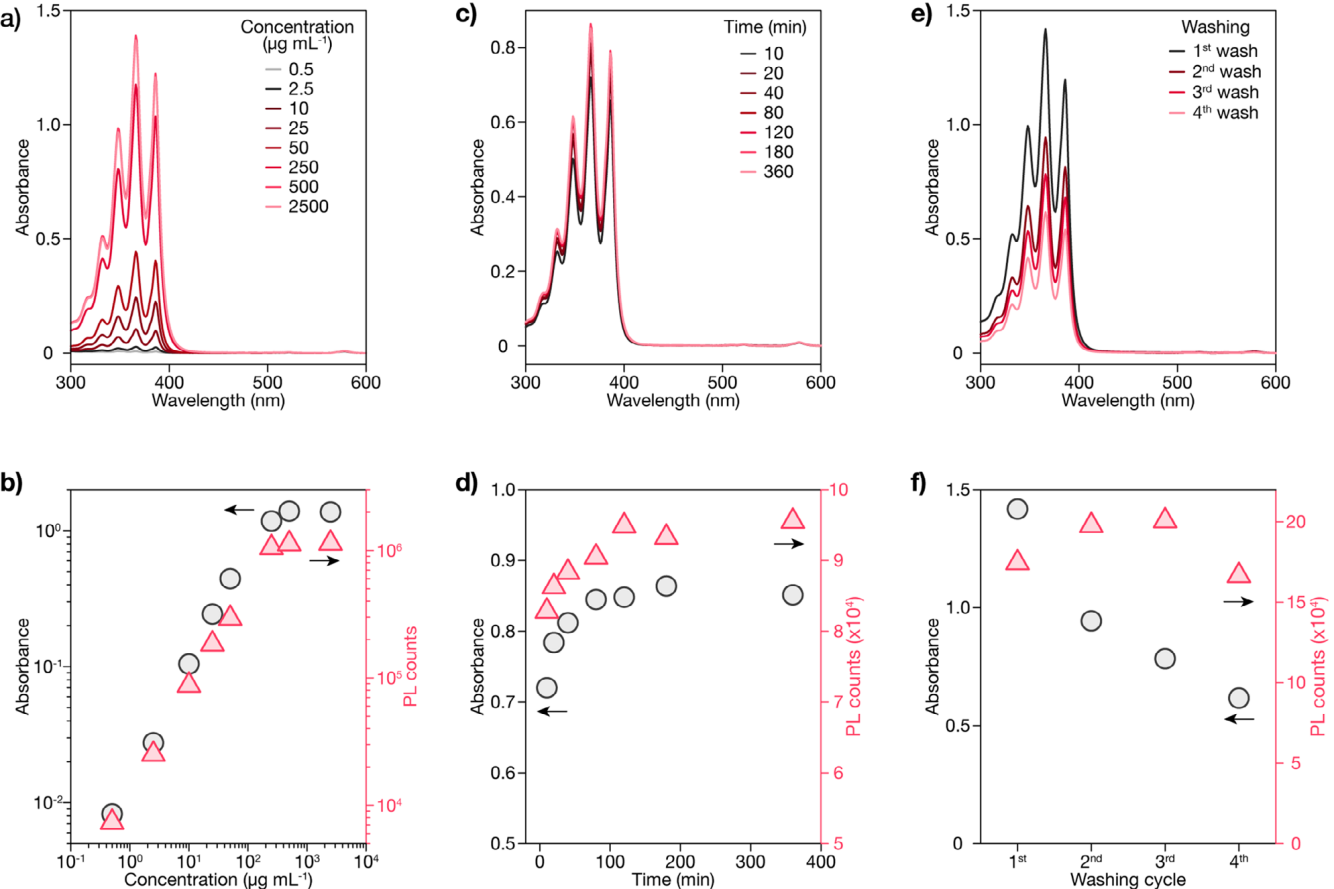
Optimization of ligand exchange conditions for ACA functionalization. a) Absorption spectra of CS_3_ NPs after ligand exchange with varying ACA concentrations (0.5–2500 µg mL^−1^). b) Corresponding absorbance at 366 nm and PL emission intensity at 980 nm as a function of ligand concentration. c) Time‐dependent absorption spectra during ligand exchange with 250 µg mL^−1^ ACA. d) Evolution of absorbance at 366 nm and PL emission intensity at 980 nm during the exchange process. e) Absorption spectra of ligand‐exchanged NPs after different numbers of washing cycles. f) Absorbance at 366 nm and PL emission intensity at 980 nm as a function of washing cycles.

We first investigated the effect of ACA concentration on ligand exchange efficiency. CS_3_ NPs at a fixed concentration (around 20 mg mL^−1^) were mixed with equal volumes of ACA solutions ranging from 0.5 to 2500 µg mL^−1^, followed by 2 h reaction at room temperature. After triple washing with ethanol, the samples were redispersed in hexane for optical characterization. Figures 3a and S10 show the absorption spectra as a function of ligand concentration, revealing progressive enhancement of the characteristic ACA absorption bands around 366 nm. As shown in Figure 3b, both the absorbance at 366 nm and PL emission intensity at 980 nm under 366 nm excitation exhibit linear growth with increasing ACA concentration. Beyond 250 µg mL^−1^, both signals plateau.

Next, we examined the kinetics of ligand exchange by monitoring the time‐dependent evolution at the optimal concentration of 250 µg mL^−1^. Figures 3c,d and S11 demonstrate that ligand exchange is relatively rapid, with both absorption and emission reaching equilibrium within 1 h.

We also evaluated the impact of washing cycles on ligand retention and optical performance. While maintaining the ligand concentration at 250 µg mL^−1^ and 1 h reaction, we varied the number of washing cycles from 1 to 4. Figures 3e,f and S12 reveal complex trends, although ligand absorption decreases progressively with increased washing, the emission intensity exhibits non‐monotonic behavior. The second wash slightly enhances emission despite reduced absorption, suggesting removal of free or loosely bound dye molecules that absorb excitation light but fail to transfer energy efficiently to the NPs. After three washes, emission stabilizes, indicating optimal removal of free dye while retaining surface‐bound sensitizers. However, the fourth wash causes noticeable emission decrease, likely due to partial removal of the bound ligands. This dynamic equilibrium reflects the reversible nature of ligand exchange, where ethanol addition during washing can perturb the ligand‐surface binding equilibrium. Based on these systematic studies, we established optimal conditions of 250 µg mL^−1^ ligand concentration, 1 h reaction time, and triple washing for ligand exchange and subsequent experiments.

### 2.4 Cascade TET in Organic–Inorganic Heterostructures

To investigate the cascade TET mechanism in our organic–inorganic heterostructures, we measured the absorption, PL excitation, PL decay, and TA spectra of the LnNP‐ACA nanohybrids. The absorption spectra showed nearly identical absorption at 980 nm (Figure S13) as NP concentrations were pre‐calibrated to ensure equivalent amounts of emitting centers for accurate comparison. Absorption in the 300–400 nm range progressively increases from C‐ACA to CS_6_‐ACA, likely due to enlarged surface area available for ACA attachment as shell thickness increases.

**FIGURE 4:**
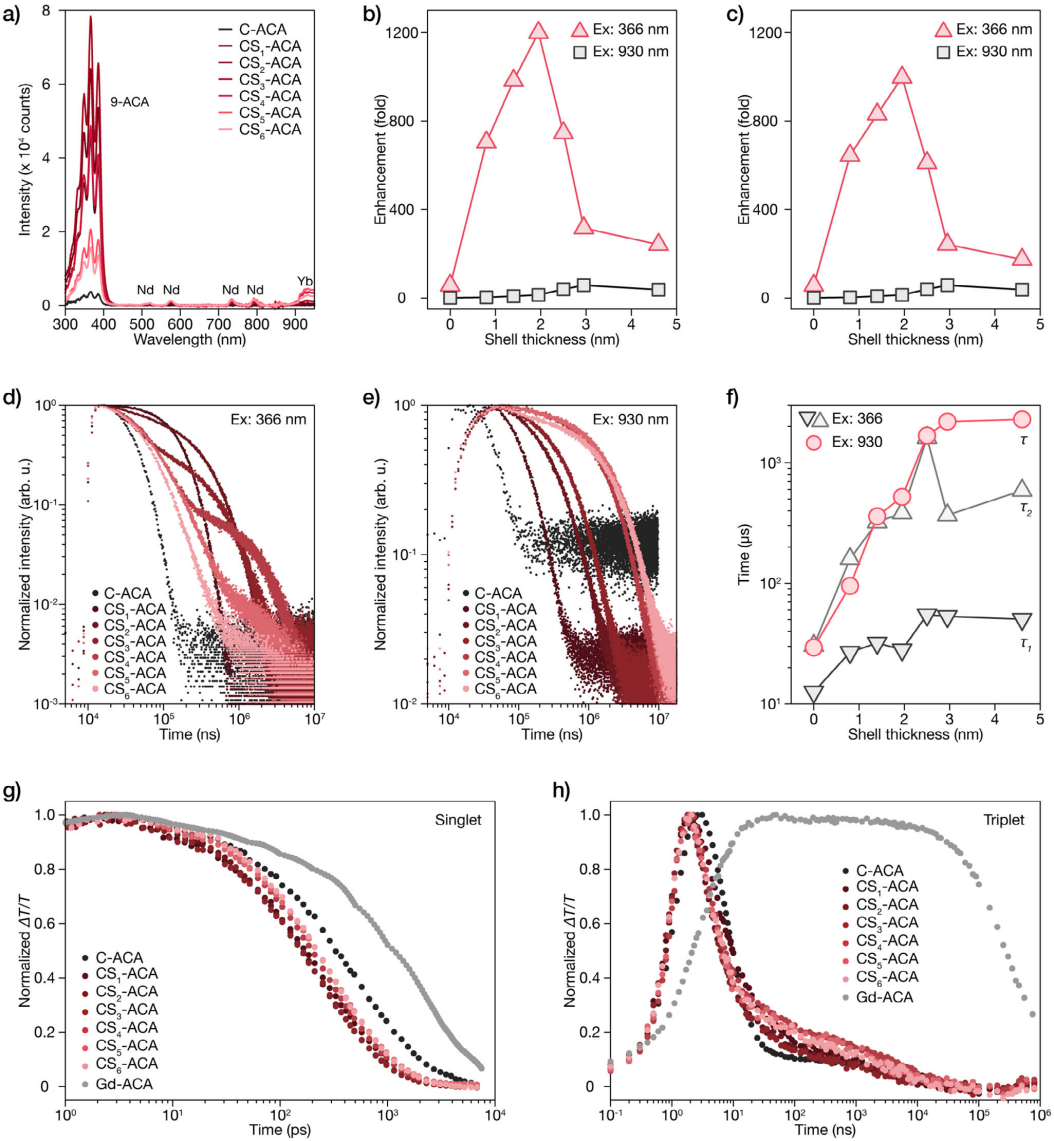
Photophysics in core/active‐shell LnNP‐ACA nanohybrids. a) Excitation spectra of NPs‐ACA hybrids monitored at Yb^3+^ emission (980 nm), calibrated by Yb^3+^ absorption at 980 nm. b) Emission enhancement as a function of shell thickness under ACA excitation (366 nm) and direct Yb^3+^ excitation (930 nm), normalized to C‐ACA emission under 930 nm excitation. c) Emission enhancement after additional normalization by ACA absorption (366 nm) to isolate energy transfer efficiency from absorption effects. d) Photoluminescence decay curves of Yb^3+^ emission in NPs‐ACA hybrids under ACA excitation (366 nm). e) Photoluminescence decay curves under direct Yb^3+^ excitation (930 nm). f) Fitted time constants from single‐exponential and bi‐exponential fitting of decay curves in d) and e) respectively. g) Picosecond transient absorption data showing normalized singlet decay kinetics. h) Nanosecond transient absorption traces of triplet decay kinetics.

Excitation spectra monitored at Yb^3+^ emission for the NP‐ACA series (Figure 4a) reveal dominant excitation peaks in the 300–400 nm range, corresponding to ACA absorption. These peaks significantly outweigh direct Yb^3+^ excitation bands, demonstrating the effectiveness of our cascade TET pathway from organic chromophores to LnNPs. The excitation spectra were recorded using a calibrated Xe lamp with intensity correction across the entire wavelength range, ensuring that the measured excitation intensities at different wavelengths reflect comparable emission outputs under equivalent excitation intensity. Figure 4b presents quantitative emission enhancement (Figure S14) versus shell thickness for both direct Yb^3+^ excitation (930 nm) and ACA excitation (366 nm). Under direct Yb^3+^ excitation, we observe a ∼50‐fold enhancement with increasing shell thickness, consistent with bare NPs (Figure 2c). In contrast, under ACA excitation, emission enhancement reaches a remarkable 1200‐fold increase as Ca_0.8_F_2_:Nd_0.2_ shell thickness increases from 0 to 2 nm, followed by gradual decrease with further thickening. This extraordinary enhancement demonstrates the power of our cascade TET mechanism operating within the heterostructure, with surface passivation (12.6‐fold for CS_3_), providing the foundation and molecular sensitization (∼95‐fold) serving as the dominant factor. The ACA molecules serve as efficient light harvesters [37, 38], while the Nd^3+^‐doped active shell enables efficient triplet energy relay to core Yb^3+^ emitters. The non‐monotonic dependence on shell thickness reflects the optimization of cascade energy transfer pathways: without shell, direct ACA → Yb^3+^ transfer suffers from surface quenching; at optimal thickness (∼2 nm), the Nd^3+^ intermediates facilitate maximum cascade TET efficiency; beyond this point, extended energy migration distances through thicker shells reduce transfer efficiency.

To account for ACA loading variations among samples, we further normalized the enhancement data by ACA absorption at 366 nm (Figures 4c and S13a). This normalization isolates energy transfer efficiency from absorption effects, allowing assessment of intrinsic energy transfer capability for each system. The normalized data maintain the same trend as unnormalized results, confirming that even with equivalent molecular loading per nanoparticle, a remarkable 1000‐fold emission enhancement can be achieved with optimal shell thickness. This underscores the important role of active shell engineering in enabling highly efficient cascade TET within our organic–inorganic heterostructures.

Figures 4d,e and S15 present Yb^3+^ emission decay curves at 980 nm under 366 nm (ACA excitation) and 930 nm (direct Yb^3+^ excitation), respectively. Under ACA excitation (Figure 4d), decay profiles exhibit bi‐exponential characteristics. These curves closely resemble those from bare NPs under 575 nm excitation (Figure 2e), confirming our proposed cascade energy transfer mechanism: excitation at 366 nm initiates the ACA → Nd^3+^ → Yb^3+^ cascade pathway, where shell Nd^3+^ ions serve as energy intermediates between organic sensitizers and core emitters. Since both pathways involve the Nd^3+^ → Yb^3+^ energy transfer step, the decay dynamics thus exhibit similar characteristics. In contrast, decay curves under direct Yb^3+^ excitation at 930 nm (Figure 4e) show monotonic evolution with increasing shell thickness, closely resembling bare NPs under the same excitation (Figure 2d). This similarity confirms that direct excitation bypasses the cascade mechanism and predominantly reflects the intrinsic core‐shell heterostructure properties.

Figure 4f quantifies the decay components extracted from fitting. Under 930 nm direct excitation, single‐exponential fitting yields time constants that increase with shell thickness, reaching 2.3 ms for the thickest shells. Under ACA excitation (366 nm), bi‐exponential fitting was required due to the multi‐step cascade energy transfer pathway, yielding fast (τ_1_) and slow (τ_2_) decay components. The decay constants are consistently shorter than those under direct Yb^3+^ excitation. Moreover, the decay dynamics show that τ_1_ progressively dominating as shell thickness increases, eventually accounting for over 90% beyond 2.5 nm (Table S2), correlating with the onset of decreased emission enhancement.

To better understand the cascade TET pathways, we performed TA spectroscopy on LnNP‐ACA nanohybrids. Figure 4g shows picosecond TA traces with normalized singlet (375 nm) signals, revealing a clear distinction between the core sample (C‐ACA) and shell‐containing samples. Quantitative analysis (Table S3) shows that C‐ACA exhibits the longest singlet decay time (677.80 ps), while shell‐containing samples display significantly faster decay (323–451 ps), demonstrating that the Nd^3+^‐doped active shell dramatically enhances ISC and FRET rates compared to direct ACA‐Yb^3+^ interactions. These findings are further corroborated by time‐resolved PL measurements monitoring surface ACA emission (Figure S16). The decay profiles show clear differences between core and shell‐containing samples, with shell samples exhibiting faster decay kinetics, confirming enhanced energy transfer initiation from ACA to shell Nd^3+^ ions.

Nanosecond TA measurements (Figure 4h) of triplet signals (430 nm) provide direct evidence of the cascade TET mechanism efficiency. Shell‐containing samples show faster triplet formation (∼1.2 ns) and decay kinetics (4.4–5.1 ns) compared to C‐ACA (1.72 ns rise, 7.66 ns decay), confirming that Nd^3+^ ions in the active shell serve as efficient triplet acceptors. CS_3_‐ACA exhibits the fastest triplet decay (4.36 ns, Table S3), indicating maximum energy transfer efficiency at this optimal shell thickness, which correlates with the emission enhancement trends observed in steady‐state measurements (Figure 4b,c). Notably, the calculated TET efficiency approaches unity for all lanthanide‐containing samples, demonstrating the exceptional performance of our cascade TET pathway. For comparison, the control sample Gd‐ACA exhibits drastically prolonged singlet (1.1 ns) and triplet (297.8 µs) lifetimes due to the absence of efficient energy acceptors, confirming that the accelerated dynamics result from the engineered T_1_ → Nd^3+^ → Yb^3+^ cascade pathway. These ultrafast spectroscopic results provide evidence that our core/active‐shell heterostructure enables unprecedented cascade TET efficiency, with shell Nd^3+^ ions functioning as ideal energy intermediates that facilitate rapid triplet capture from organic sensitizers and efficient relay to core Yb^3+^ emitters, ultimately enabling the remarkable 1200‐fold emission enhancement.

## 3 Conclusion

In this work, we demonstrate a cascade TET mechanism in organic–inorganic heterostructures. Our NaYbF_4_@Ca_0.8_F_2_:Nd_0.2_@ACA heterostructure achieves a remarkable 1200‐fold emission enhancement by engineering an active shell that functions as an efficient energy relay pathway. The cascade TET operates through a precisely designed three‐step pathway, i.e., ISC in ACA generates triplet excitons, these triplets transfer to shell Nd^3+^ ions via Dexter‐like interactions, and energy subsequently migrates to core Yb^3+^ ions. This heterostructure approach differs from FRET‐based designs by exploiting the superior efficiency of triplet energy transfer, approaching 100% as confirmed by TA spectroscopy. The Nd^3+^ ions serve as critical intermediates, exhibiting optimal spectral overlap with both ACA triplet states (∼1.83 eV) [34] and Yb^3+^ excited state, enabling efficient triplet transportation across the inorganic shell barrier.

Our investigation demonstrates that heterostructure design enables precise control over cascade TET efficiency through shell thickness optimization. Both key steps (T_1_ → Nd^3+^ and Nd^3+^ → Yb^3+^) of the cascade mechanism can achieve near‐unity efficiency within our heterostructure. However, the overall cascade performance depends critically on shell thickness. While individual transfer steps remain highly efficient, excessive shell thickness introduces extended energy migration pathways that increase non‐radiative losses. The optimal thickness (∼2.0 nm) represents the critical design parameter where the cascade TET mechanism achieves maximum overall efficiency. Beyond this threshold, the cumulative effect of longer migration distances begins to compromise the performance of the cascade mechanism.

These findings establish design principles for cascade TET systems: heterogeneous core‐shell compositions prevent cation intermixing while ensuring crystallographic compatibility, active shells should incorporate ions with optimal triplet spectral overlap, shell thickness must be precisely engineered to maximize cascade efficiency. Our cascade TET strategy demonstrates that organic–inorganic heterostructures can achieve unprecedented performance through rational design of energy transfer pathways, providing a platform for next‐generation lanthanide‐based materials in bioimaging, photon upconversion, and optoelectronic devices.

## Conflicts of Interest

The authors declare no conflicts of interest.

## Supporting information




**Supporting File 1**: anie71602‐sup‐0001‐SuppMat.docx.

## Data Availability

The data underlying all figures in the main text are publicly available from the University of Cambridge repository at https://doi.org/10.17863/CAM.127529.
