# Supplementary material for: Active Shell Engineering for Efficient Cascade Triplet Energy Transfer in Lanthanide Heterostructures
Source: Angew Chem Int Ed Engl. 2026 Feb 22;65(14):e2017963. doi: 10.1002/anie.2017963 (PMC13023699; doi:10.1002/anie.2017963)
Supplement: Supplementary file 1 — Supporting File 1: anie71602‐sup‐0001‐SuppMat.docx. [file ANIE-65-e2017963-s001.docx]

**Supporting Information**

Active Shell Engineering for Efficient Cascade Triplet Energy Transfer in Lanthanide Heterostructures

Zhao Jiang,^[a]^ Alasdair Tew,^[a]^ Xinjuan Li,^[b]^ Huangtianzhi Zhu,^[a]^ Yunzhou Deng,^[a]^ Caterina Ducati,^[b]^ Zhongzheng Yu,*^[a]^ Akshay Rao*^[a]^

[a] Z. Jiang, A. Tew, H. Zhu, Y. Deng, Z. Yu, A. Rao

Cavendish Laboratory
University of Cambridge
Cambridge, CB3 0HE, United Kingdom
E-mail: zy338@cam.ac.uk (Z. Yu), ar525@cam.ac.uk (A. Rao)

[b] X. Li, C. Ducati
Department of Materials Science and Metallurgy
University of Cambridge
Cambridge, CB3 0FS, United Kingdom

**Supporting Methods**

**Chemicals and Materials**

Ytterbium acetate hydrate (Yb(CH_3_CO_2_)_3_·xH_2_O, 99.9%), sodium hydroxide (NaOH, >98%), ammonium fluoride (NH_4_F, 99%), calcium carbonate (CaCO_3_, 99%), neodymium(III) oxide (Nd_2_O_3_, 99.9%), trifluoroacetic acid (TFA, 99%), 1-octadecene (ODE, 90%), oleic acid (OA, 90%), 9-anthracenecarboxylic acid (9-ACA, 99%), and all anhydrous solvents (hexane, ethanol, etc.) were purchased from Sigma-Aldrich. All chemicals were used as received without further purification.

**Synthesis of α-NaYbF_4_ core nanoparticles**

1.0 mmol of Yb(CH_3_CO_2_)_3_ was added to a 50 mL flask containing OA (6 mL) and ODE (14 ml). The mixture was heated to 140 °C and maintained for 30 min under N_2_ flow and then cooled down to room temperature. Subsequently, a methanol solution (6 mL) containing NH_4_F (0.72 mmol) and NaOH (0.45 mmol) was added and stirred at 70 °C for 45 min to remove the residual methanol from the reaction mixture. Upon the removal of methanol, the solution was heated to 280 °C and maintained at this temperature under a nitrogen flow for 30 min. The solution was then cooled down to room temperature naturally. The resulting nanoparticles were precipitated out with an addition of ethanol, collected by centrifugation, and finally dispersed in 6 ml of hexane.

**CaF_2_:20%Nd^3+^ shelling of core nanoparticles**

**Preparation of calcium trifluoroacetate, Ca(TFA)_2_.** CaCO_3_ (5 g) was added to a 50 mL flask. To the flask was slowly added excess water and trifluoroacetic acid (1:1). The mixture was then stirred at 80  °C until the solution became completely clear and transparent. After cooling to room temperature, this solution was filtered. Finally, the solvents of water and trifluoroacetic acid were evaporated under reduced pressure. A white dry powder of Ca(TFA)_2_ could be obtained with a yield of >90%.

**Preparation of neodymium trifluoroacetate, Nd(TFA)_3_.** Neodymium trifluoroacetate was prepared by dissolving Nd_2_O_3_ in trifluoroacetic acid using the similar protocols as above.

**CaF_2_:20%Nd^3+^ shelling.** 0.25 mmol of NaYbF_4_ core NPs and designed moles of shell precursors were added to a 50 mL flask containing OA (15 mL) and ODE (15 ml). The mixture was heated to 120 °C and evacuated for 20 min. Then the solution was heated to 300 °C under nitrogen flow and maintained for 30~90 min. Then the solution was cooled to room temperature, and the NPs were purified three times with ethanol and hexane, and finally dispersed in hexane. The amounts of shell precursor were 0.2, 0.4, 0.6, 1.0, 2.0, 3.0 for CS1 to CS6 NPs, respectively.

**TEM and optical characterizations**

Transmission electron microscopy (TEM) was performed using an FEI Tecnai F20 at 200 kV accelerating voltage. High-resolution HAADF-STEM was performed on a TF Spectra 300 (200 kV, 17.1 mrad convergence angle, 115 mm camera length, 1 μs dwell time, 1 pA current). Collection angles: 74.3 ± 0.3 to 233.3 ± 1.5 mrad. Images were acquired at 512 × 512 pixels over 10 frames with a 1 μs pixel dwell time. Drift-corrected frame integration (DCFI) was applied to reconstruct a periodic image, enhancing spatial resolution. Post-acquisition, a radial and high-pass filter was applied to the reconstructed image to enhance sub-angstrom feature visibility and improve the contrast of light-element columns. Energy Dispersive X-ray Spectroscopy (STEM-EDX) maps were acquired using four Super-X detectors with a dwell time of 200 μs per pixel across 100 frames, achieving a spectral resolution of 10 eV per channel. STEM-EDX spectrum images were collected using a beam current of 30 pA, a convergence angle of 23.4 mrad, and a camera length of 115 mm. For optical measurements, samples were prepared in a nitrogen-atmosphere glovebox using degassed, anhydrous solvents. Cuvettes were sealed with a PTFE cap, PTFE tape, and parafilm, to ensure the absence of oxygen inside the samples. Steady-state absorption spectra were collected by a Shimadzu UV3600Plus spectrometer. Steady-state photoluminescence spectra were measured using an Edinburgh Instruments FLS1000 spectrometer, equipped with xenon lamp for excitation. The PL decay curves of Yb were also measured by FLS1000 spectrometer but using a micfrosecond flashlamp as excitation source. The fluorescence decay curves of 9-ACA were measured using a time-correlated single photon counting (TCSPC) setup, equipped with a 375-nm pulsed laser (pulse width <200 ps, repetition rate: 40 MHz) for excitation and a silicon-based single-photon avalanche photodiode for photon detection. The instrument response function has a lifetime of less than 0.2 ns.

**Transient absorption spectroscopy**

Picosecond transient absorption (TA) measurements were made using a system from Light Conversion consisting of a PHAROS 1030 nm fundamental laser at a 10 kHz repetition rate, an ORPHEUS OPA for pump generation (355 nm) and the HARPIA TA system for white light generation, delay and capture. For picosecond measurements the pump power was 2.5 mW with a beam diameter of 1360 um and a repetition rate of 10 kHz giving a total energy per pulse of 17 uJ cm^–2^.

Nanosecond transient absorption measurements were made using a home built setup consisting of a white light super continuum probe laser from Leukos (DISCO) and a 355 nm pump laser from Innolas (Picolo). Delay between the pump and probe was digitally generated using Highland Technology T560 delay generators. A Streising line camera system was used capture both probe and reference. A function generator from Agilent alongside a Stanford instruments SRS DG645 were used to prepare and shape the initial electronic pulses from triggering the lasers and camera system. For nanosecond measurements the pump power used was 0.5 mW with a beam diameter of 420 um and repetition rate of 1 kHz giving a total energy per pulse of 0.36 mJ cm^–2^.

**Supporting Figures and Tables**


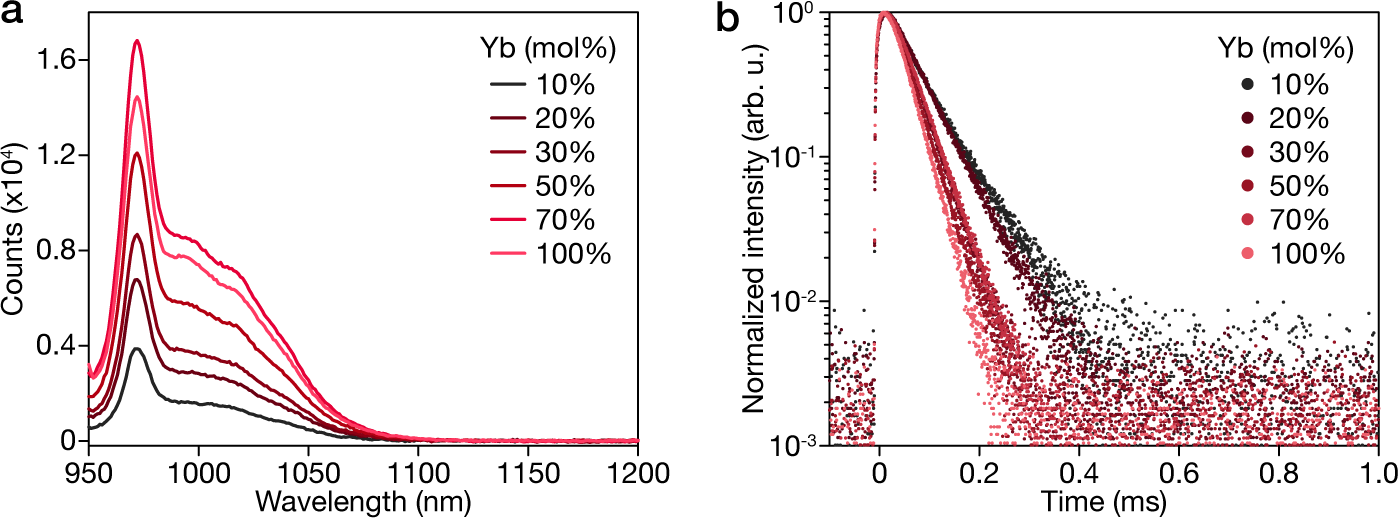


**Figure S1.** (a) Emission spectra of NaGdF_4_:x%Yb^3+^ (x=10, 20, 30, 50, 70, 100). (b) Corresponding PL decay curves of NaGdF_4_:x%Yb^3+^ (x=10, 20, 30, 50, 70, 100). Excitation: 930 nm.


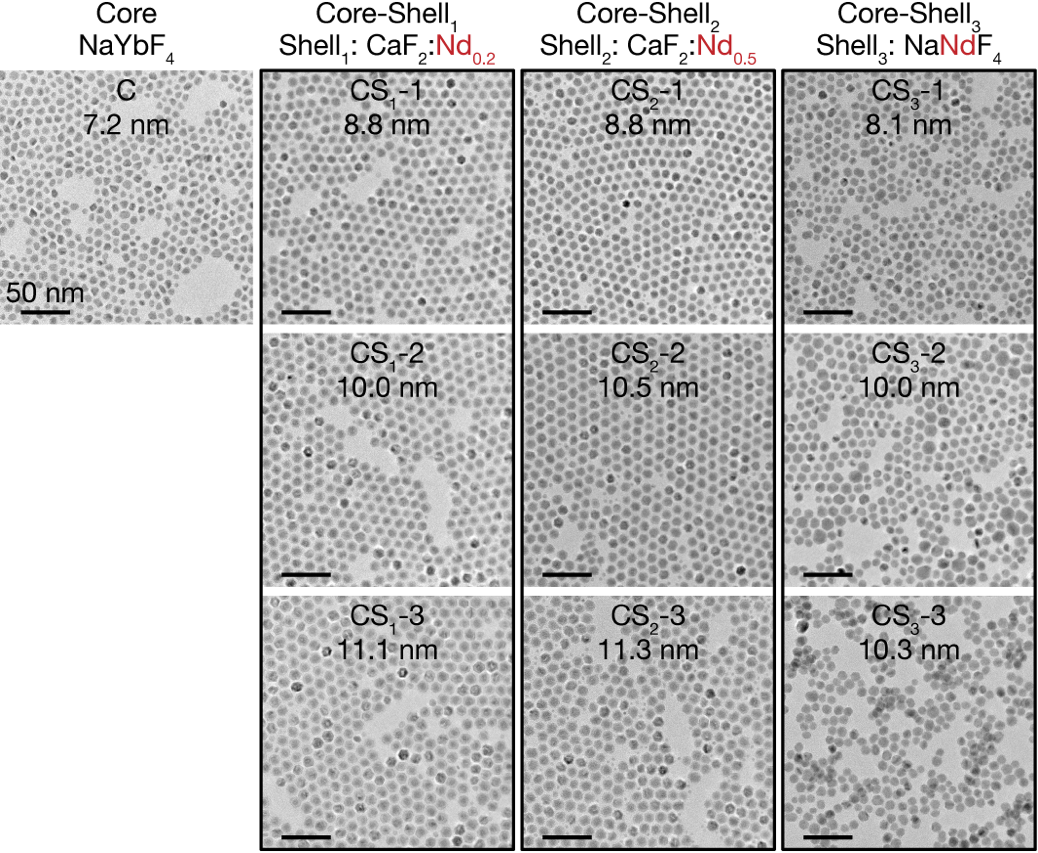


**Figure S2.** TEM images of NaYbF_4_@Ca_1–x_F_2_:Nd_x_ core-shell nanoparticles with varying Nd^3+^ doping concentrations (x = 0.2, 0.5, 1.0) and shell thicknesses.


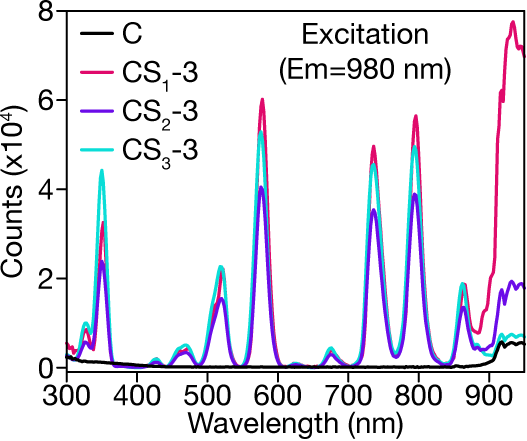


**Figure S3.** Photoluminescence excitation spectra of NaYbF_4_@Ca_1–x_F_2_:Nd_x_ core-shell nanoparticles with x = 0.2, 0.5, and 1.0, by monitoring Yb^3+^ emission at 980 nm.


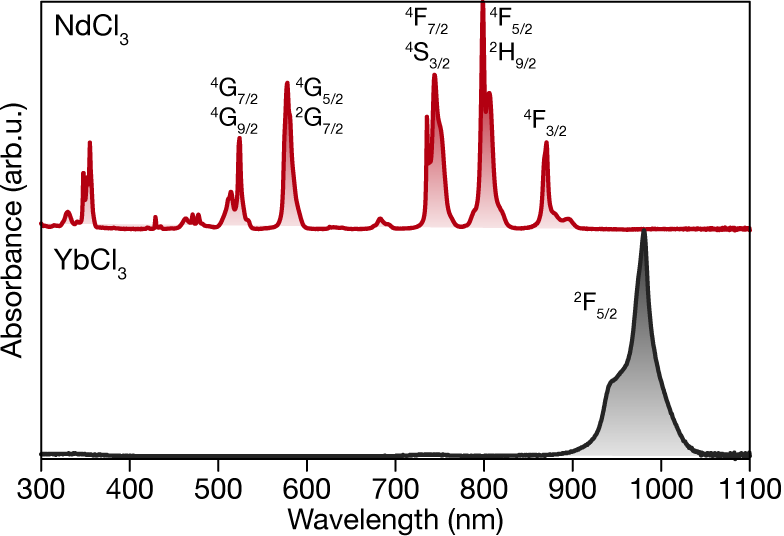


**Figure S4.** Normalized absorption spectra of NdCl_3_ and YbCl_3_ in aqueous solution.


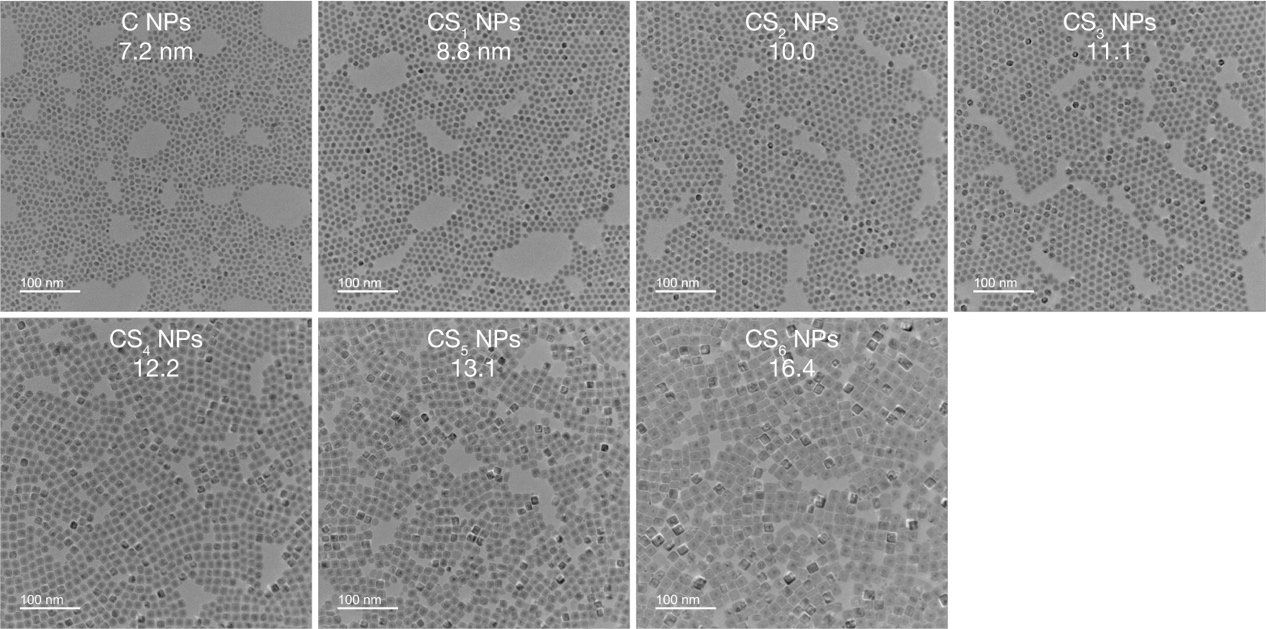


**Figure S5.** TEM images of NaYbF_4_ core and NaYbF_4_@CaF_2_:Nd_0.2_ core-shell NPs.


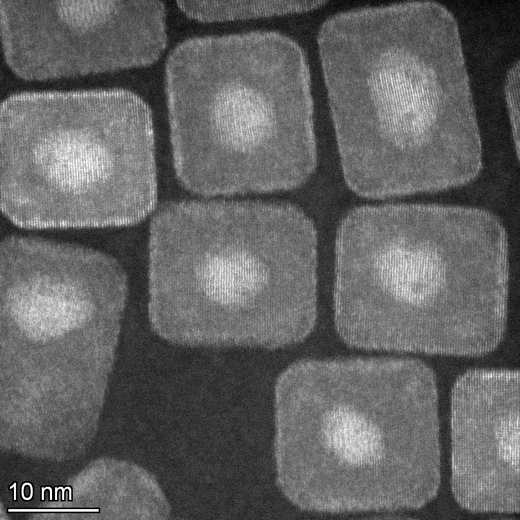


**Figure S6.** HAADF-STEM image of sample CS_6_.

**Table S1.** Lattice planes and corresponding d-spacings of NaYbF_4_ (*Fm*-3*m*) and CaF_2_ (*Fm*-3*m*).

| NaYbF_4_ (*Fm*-3*m*) | | CaF_2_ (*Fm*-3*m*) | |
| --- | --- | --- | --- |
| ( h k l ) | d (Å) | ( h k l ) | d (Å) |
| ( 1 1 1 ) | 3.1263 | ( 1 1 1 ) | 3.1546 |
| ( 2 0 0 ) | 2.7075 | ( 2 0 0 ) | 2.7314 |
| ( 2 2 0 ) | 1.9145 | ( 2 2 0 ) | 1.9316 |
| ( 3 1 1 ) | 1.6327 | ( 3 1 1 ) | 1.6471 |
| ( 2 2 2 ) | 1.5632 | ( 2 2 2 ) | 1.5771 |
| ( 4 0 0 ) | 1.3538 | ( 4 0 0 ) | 1.3656 |


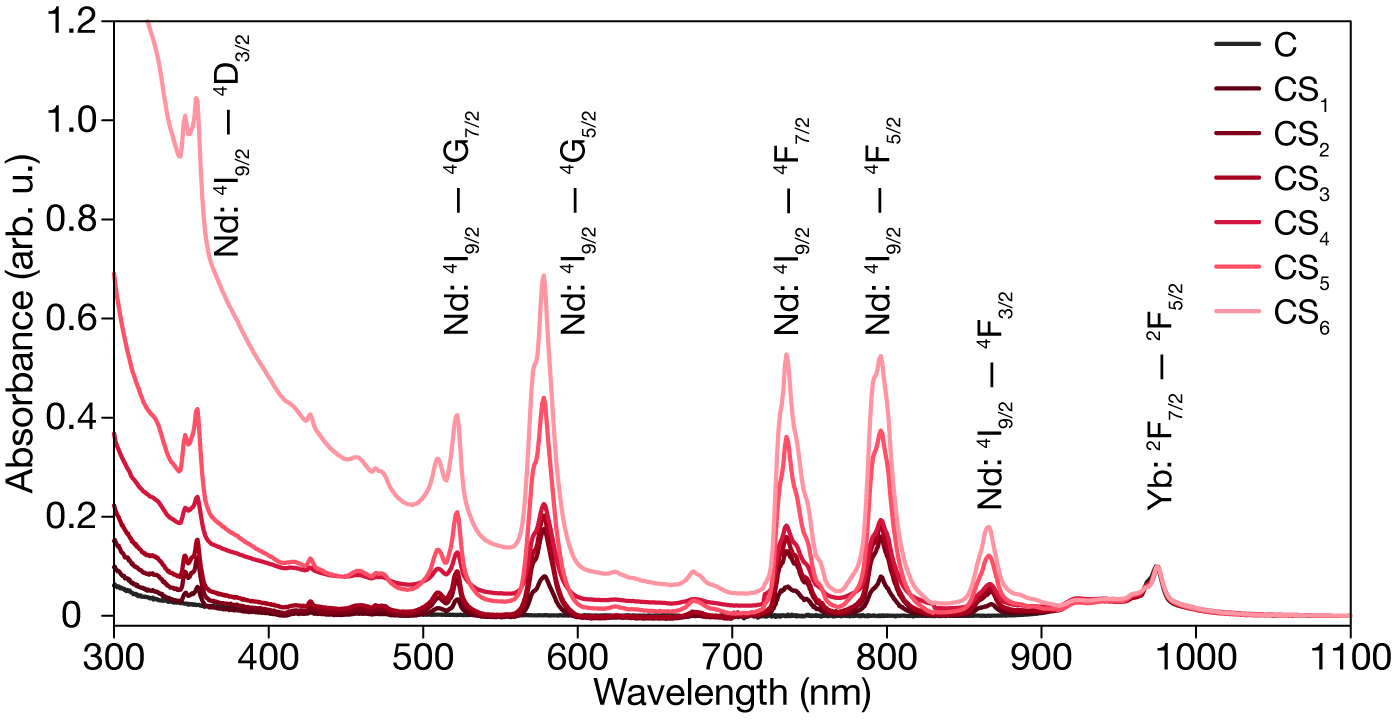


**Figure S7.** Absorption spectra of core and core-shell NPs.


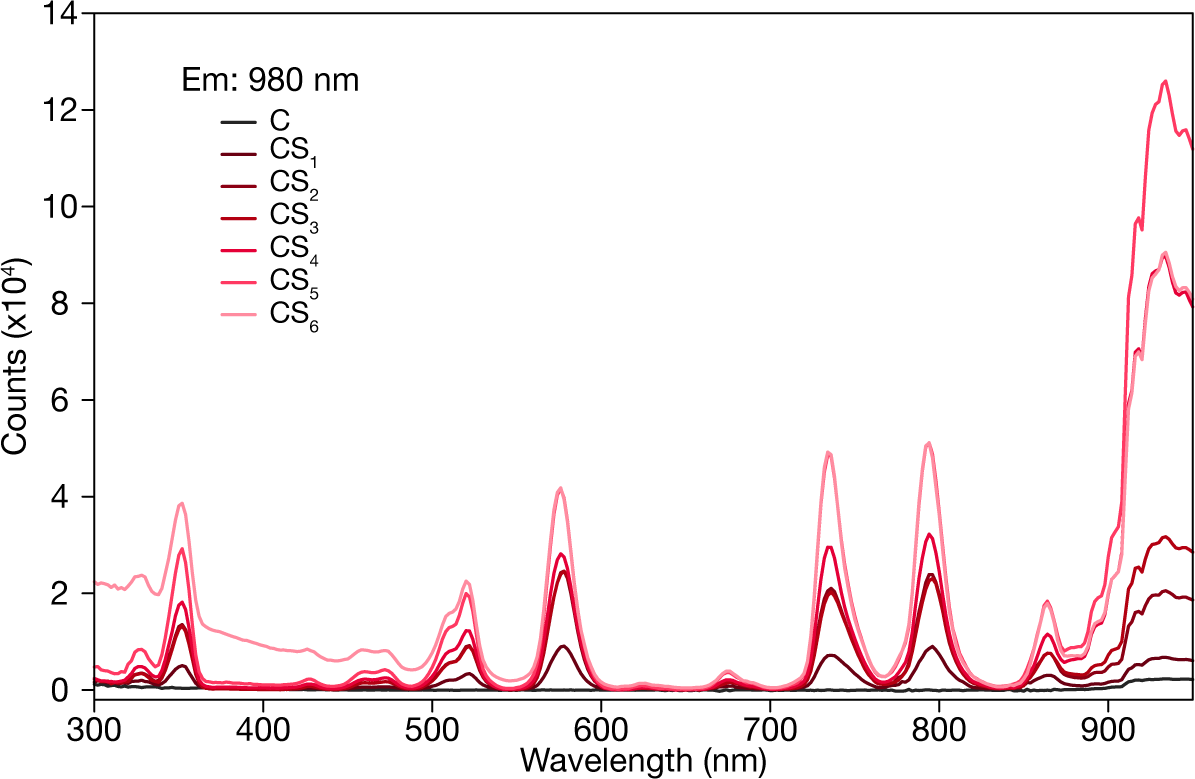


**Figure S8.** Excitation spectra of NaYbF_4_ core and NaYbF_4_/CaF_2_:Nd_0.2_ core-shell NPs by monitoring the Yb^3+^ emission at 980 nm.


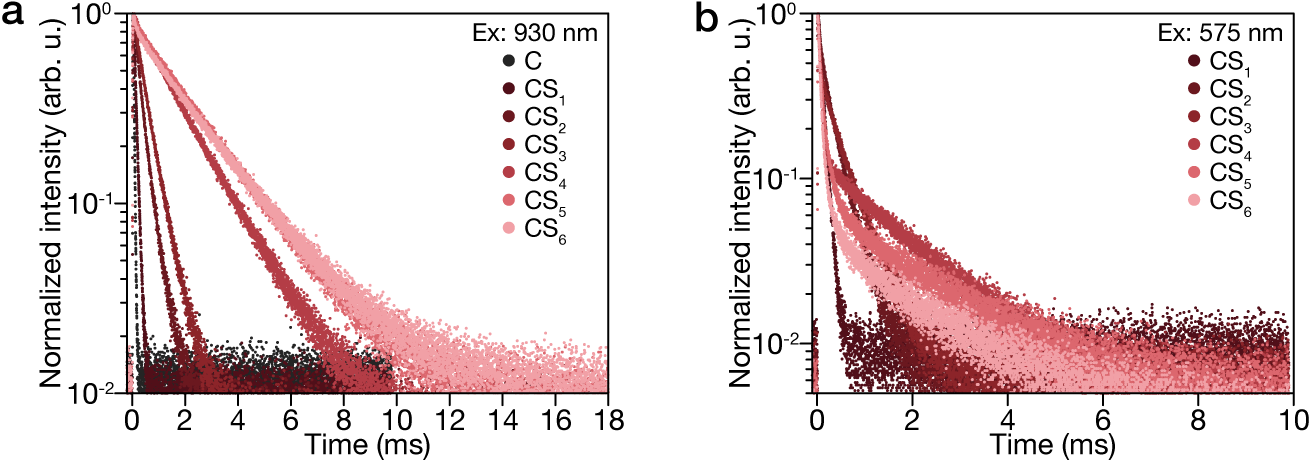


**Figure S9.** (a) Photoluminescence decay curves of Yb^3+^ emission at 980 nm under 930 nm excitation of Yb^3+^. (b) Photoluminescence decay curves of Yb^3+^ emission at 980 nm under 575 nm excitation of Nd^3+^.

**Table S2.** Extracted time constants (fast component: τ_1_, slow component: τ_2_) and their relative weights (w_1_ and w_2_) using single-exponential (for 930 nm excitation) and bi-exponential (for 575 and 366 nm excitation) models.

|  | NPs (Ex 930 nm) | | NPs (Ex 575 nm) | | | |
| --- | --- | --- | --- | --- | --- | --- |
| Shell thickness (nm) | τ (μs) | w (%) | τ_1_ (μs) | w_1_ (%) | τ_2_ (μs) | w_2_ (%) |
| 0.00 | 41.95 | 100.00 |  |  |  |  |
| 0.80 | 99.32 | 100.00 | 15.56 | 0.00 | 100.35 | 100.00 |
| 1.40 | 371.48 | 100.00 | 56.90 | 39.80 | 325.04 | 60.20 |
| 1.95 | 545.24 | 100.00 | 37.51 | 58.87 | 466.41 | 41.13 |
| 2.50 | 1720.74 | 100.00 | 50.80 | 88.85 | 1888.97 | 11.15 |
| 2.95 | 2194.46 | 100.00 | 63.50 | 87.71 | 628.26 | 12.29 |
| 4.60 | 2289.21 | 100.00 | 51.52 | 93.04 | 1289.78 | 6.96 |
|  |  |  |  |  |  |  |
|  | NPs-ACA (Ex 930 nm) | | NPs-ACA (Ex 366 nm) | | | |
| Shell thickness (nm) | τ (μs) | w (%) | τ_1_ (μs) | w_1_ (%) | τ_2_ (μs) | w_2_ (%) |
| 0.00 | 29.38 | 100.00 | 12.57 | 0.00 | 30.45 | 100.00 |
| 0.80 | 95.24 | 100.00 | 26.81 | 0.00 | 159.28 | 100.00 |
| 1.40 | 359.25 | 100.00 | 32.00 | 21.31 | 321.79 | 78.69 |
| 1.95 | 521.37 | 100.00 | 28.14 | 66.37 | 382.71 | 33.63 |
| 2.50 | 1680.59 | 100.00 | 54.90 | 91.64 | 1611.58 | 8.36 |
| 2.95 | 2193.96 | 100.00 | 53.19 | 88.81 | 367.63 | 11.19 |
| 4.60 | 2289.84 | 100.00 | 50.40 | 93.80 | 588.71 | 6.20 |


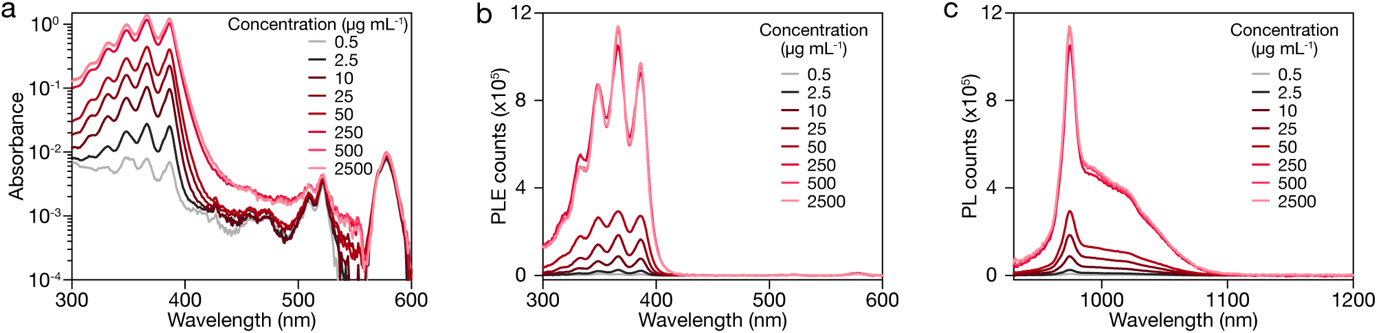


**Figure S10.** (a) Absorption, (b) PL excitation, (c) PL emission spectra of CS_3_ NPs after ligand exchange with varying 9-ACA concentrations (0.5-2500 μg mL^–1^).


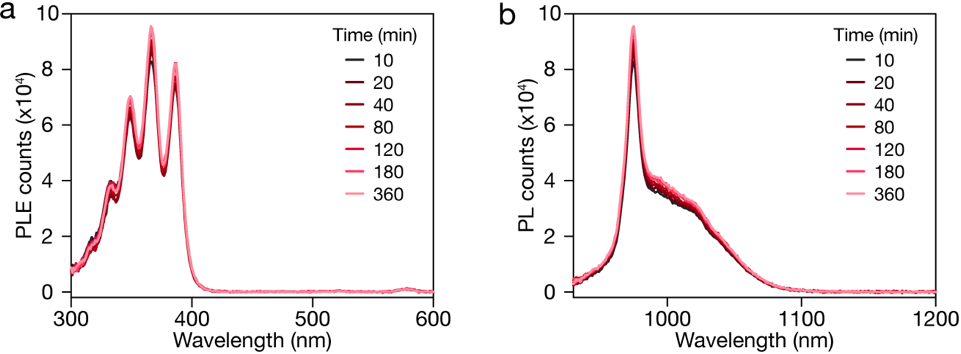


**Figure S11.** Time-dependent (a) PL excitation and (b) PL emission spectra during ligand exchange with 250 μg mL^–1^ 9-ACA.


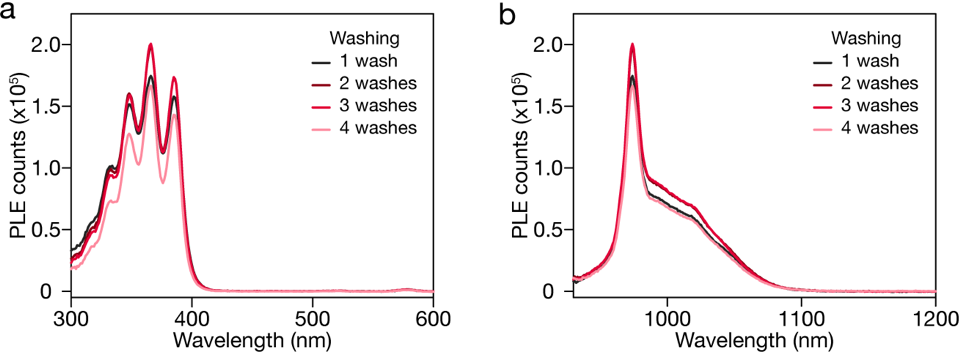


**Figure S12.** (a) PL excitation and (b) PL emission spectra of ligand-exchanged NPs after different numbers of washing cycles.


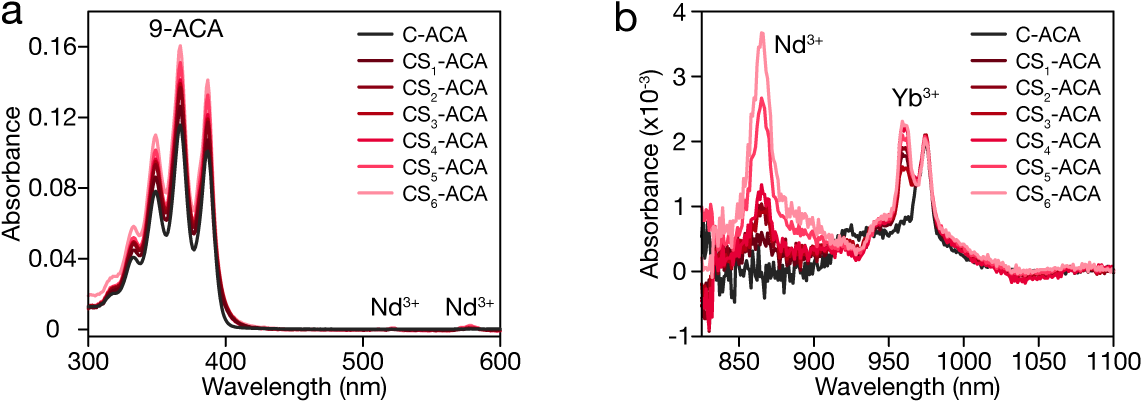


**Figure S13.** Absorption spectra of NP-ACA samples at UV-VIS range (a) and NIR range (b), respectively.


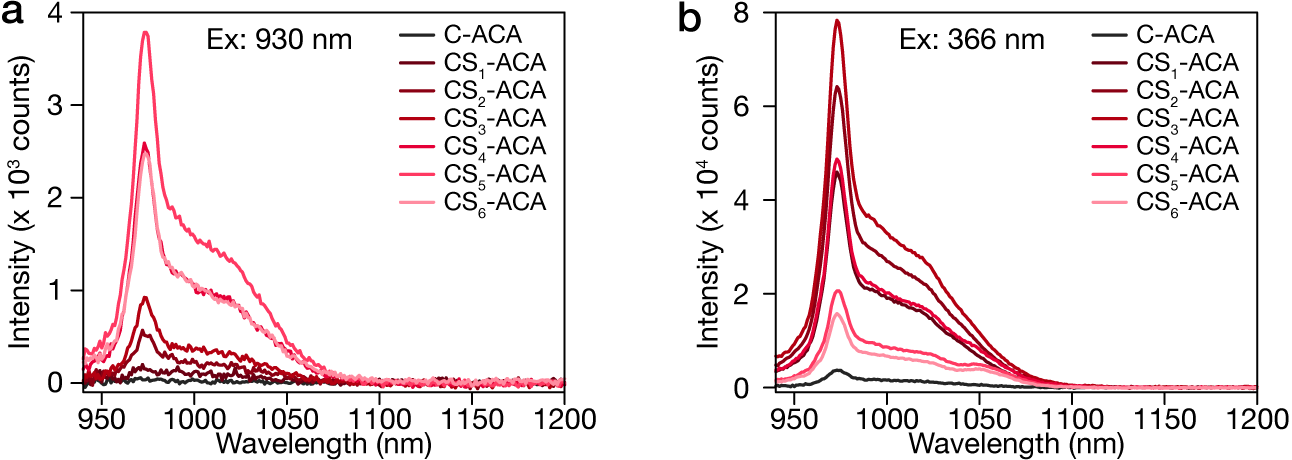


**Figure S14.** Emission spectra as a function of shell thickness under (a) ACA excitation (366 nm) and (b) direct Yb^3+^ excitation (930 nm), without normalization.


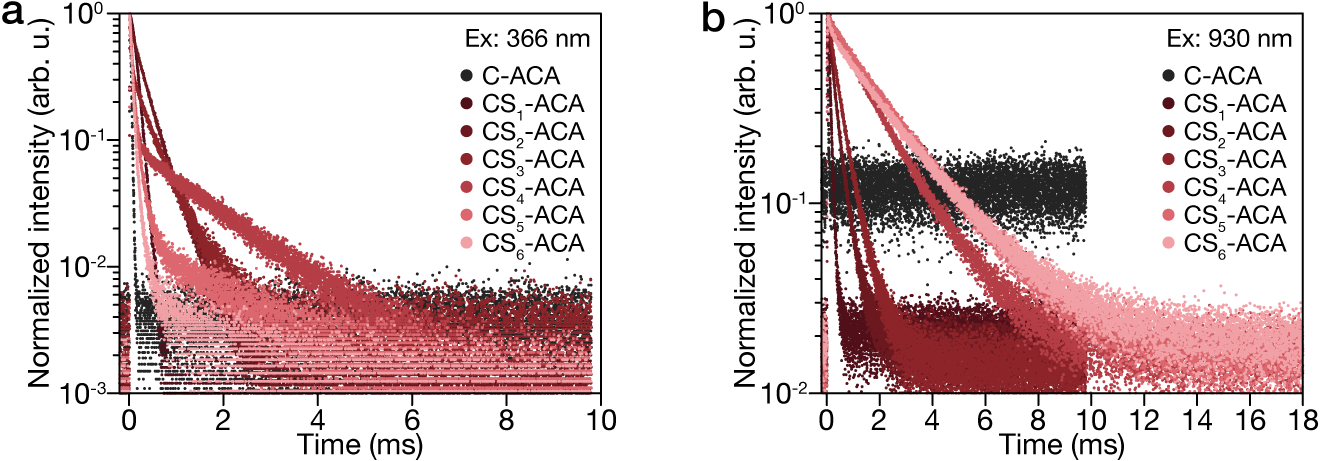


**Figure S15.** (a) Photoluminescence decay curves of Yb^3+^ emission in NPs-ACA hybrids under ACA excitation (366 nm). (b) Photoluminescence decay curves under direct Yb^3+^ excitation (930 nm).

**Table S3.** Fitted average lifetime and calculated TET efficiency from TA spectra.

|  | Singlet decay (ps) | Triplet rise (ns) | Triplet decay (ns) | TET efficiency |
| --- | --- | --- | --- | --- |
| C-ACA | 677.80 | 1.72 | 7.66 | 1.00 |
| CS_1_-ACA | 361.75 | 1.40 | 7.24 | 1.00 |
| CS_2_-ACA | 323.39 | 1.22 | 4.37 | 1.00 |
| CS_3_-ACA | 326.76 | 1.22 | 4.36 | 1.00 |
| CS_4_-ACA | 386.96 | 1.21 | 4.87 | 1.00 |
| CS_5_-ACA | 413.39 | 1.23 | 4.51 | 1.00 |
| CS_6_-ACA | 450.57 | 1.21 | 5.07 | 1.00 |
| Gd-ACA | 1135.83 | 5.68 | 297765.95 |  |


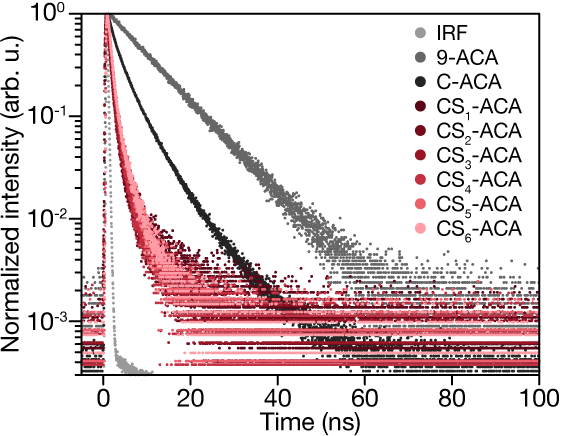


**Figure S16.** Time-resolved PL spectra by monitoring the surface 9-ACA emission of NP-ACA samples.
